# Photoinitiated Polymerization of 2-Hydroxyethyl Methacrylate by Riboflavin/Triethanolamine in Aqueous Solution: A Kinetic Study

**DOI:** 10.1155/2013/958712

**Published:** 2013-09-23

**Authors:** Iqbal Ahmad, Kefi Iqbal, Muhammad Ali Sheraz, Sofia Ahmed, Tania Mirza, Sadia Hafeez Kazi, Mohammad Aminuddin

**Affiliations:** ^1^Institute of Pharmaceutical Sciences, Baqai Medical University, 51 Deh Tor, Toll Plaza, Super Highway, Gadap Road, Karachi 74600, Pakistan; ^2^Department of Dental Material Sciences, Baqai Dental College, Baqai Medical University, 51 Deh Tor, Toll Plaza, Super Highway, Gadap Road, Karachi 74600, Pakistan

## Abstract

The polymerization of 1–3 M 2-hydroxyethyl methacrylate (HEMA) initiated by riboflavin/triethanolamine system has been studied in the pH range 6.0–9.0. An approximate measure of the kinetics of the reaction during the initial stages (*~*5% HEMA conversion) has been made to avoid the effect of any variations in the volume of the medium. The concentration of HEMA in polymerized solutions has been determined by a UV spectrophotometric method at 208 nm with a precision of ±3%. The initial rate of polymerization of HEMA follows apparent first-order kinetics and the rates increase with pH. This may be due to the presence of a labile proton on the hydroxyl group of HEMA. The second-order rate constants for the interaction of triethanolamine and HEMA lie in the range of 2.36 to 8.67 × 10^−2^ M^−1^ s^−1^ at pH 6.0–9.0 suggesting an increased activity with pH. An increase in the viscosity of HEMA solutions from 1 M to 3 M leads to a decrease in the rate of polymerization probably as a result of the decrease in the reactivity of the flavin triplet state. The effect of pH and viscosity of the medium on the rate of reaction has been evaluated.

## 1. Introduction

Acrylic acid derivatives including 2-hydroxyethyl methacrylate monomer (HEMA) [1], urethane dimethacrylate monomer [2], proline modified acrylic acid copolymer [3], N-vinylcaprolactam-containing acrylic acid terpolymer [4], N-vinylpyrrolidone modified acrylic acid copolymer [5], and polyurethane acrylate monomer [6] have been synthesized for dental cement applications. These derivatives are intended to undergo polymerization on exposure to visible light and thus form a hardened mass (cement). Among these derivatives, HEMA is widely used in glass ionomer cements (GICs) employed as dental restorative materials [7]. Various types of GICs containing HEMA have been developed as light cure restorative materials [8].

The photoinitiated polymerization of vinyl polymers has been studied since the 1950s [9–12]. The process involves the participation of photoinitiators absorbing in the visible region. Riboflavin (RF) absorbs at 444 nm and has been used as a photoinitiator in the polymerization of HEMA along with triethanolamine (TEOHA) as a coinitiator to form a redox pair involved in the process [13–16]. RF is an efficient electron acceptor and meditates in numerous photochemical and biological electron transfer reactions [17–22]. The kinetics of polymerization reactions has been discussed by several workers [12, 23–25]. The medium characteristics, ionization behavior of reacting species, and efficiency of the photoinitiators influence the rate of reaction [26]. In most cases, the polymerization rates of HEMA have been determined dilatometrically [13–15]. This method has been used to study the effect of solvent on the rates of polymerization of HEMA photoinitiated by azo compounds [26, 27], camphorquinone [28, 29], and pyrene derivatives [30]. In this work, we report a study of the approximate measure of the kinetics of polymerization of HEMA initiated by RF/TEOHA system under visible light using a spectrophotometric method for the assay of HEMA during the reactions. The effects of pH, viscosity, and TEOHA concentration on the initial rates of the reaction (~5% HEMA conversion) have been evaluated. These factors have been found to influence the kinetics of the reaction and hence the efficiency of the polymerization process. The study would provide information on the photoinitiation reaction and interaction of HEMA and RF/TEOHA system in the pH range 6.0–9.0 and would facilitate the understanding of the polymerization processes in light-cure GIC systems.

## 2. Materials and Methods

### 2.1. Chemicals

 Riboflavin (RF), lumiflavin (LF), and lumichrome (LC) were obtained from Sigma and used as received. Triethanolamine (TEOHA, Sigma) and 2-hydroxyethyl methacrylate (HEMA, Aldrich) were distilled under reduced pressure before use. Water was purified using a Millipore Milli-Q system.

### 2.2. Polymerization

Polymerization of HEMA (monomer/water ratio 1.21 : 8.79, 2.42 : 7.58 and 3.63 : 6.37, v/v, corresponding to 1, 2, and 3 M) was carried out in the presence of 1 × 10^−5^ M RF (absorbance of the solution at 444 nm was low (0.125) to avoid inhomogeneous free radical distribution) [31] and 0.0025–0.01 M TEOHA at pH 6.0–9.0 (adjusted by HCl/NaOH solution) under anaerobic conditions at 25°C. The solution was irradiated with a General Electric 15 W fluorescent lamp (emission in the visible region) fixed horizontally at a distance of 25 cm from the centre of the vessel.

### 2.3. Thin-Layer Chromatography

Thin-layer chromatography (TLC) of the polymerized solutions containing RF was carried out on 250-*μ*m cellulose plate using the solvent systems: (a) 1-butanol-acetic acid-water (40 : 10 : 50, v/v, organic phase) and (b) 1-butanol-1-propanol-accetic acid-water (50 : 30 : 2 : 18, v/v) [32]. RF and photoproducts were detected by comparison of their characteristic fluorescence emission under UV (365 nm) excitation (RF, yellow green; LC, sky blue) with those of the reference standards.

### 2.4. Spectral Measurement

All spectral measurements on fresh and polymerized solutions of HEMA were carried out on a Shimadzu UV-1601 recording spectrophotometer using quartz cells of 10 mm path length.

### 2.5. Light Intensity Measurement

The measurement of the intensity of General Electric 15 W fluorescent lamp was carried out by potassium ferrioxalate actinometry [33], and a value of 2.85 ± 0.26 × 10^16^ quanta s^−1^ was obtained.

### 2.6. Assay of HEMA

 The assay of HEMA in fresh and polymerized solutions was carried out by adjusting the pH to 7.0 (0.05 M phosphate buffer) and measurement of absorbance at 208 nm after appropriate dilution. At this dilution, the photoinitiator has negligible absorption at the analytical wavelength. The calibration data for the assay of HEMA are reported in Table 1.

### 2.7. Viscosity Measurements

The viscosity of the HEMA solutions was measured with a Brookfield RV viscometer (Model DV-II + Pro, Essex, UK).

## 3. Results and Discussion

Before a consideration of the polymerization process, it is necessary to evaluate the photostability of RF as an initiator during the reaction and whether there is any alteration in its concentration that might affect the rate of the reaction.

### 3.1. Photoproducts of RF

RF has been used by many workers as a photoinitiator in the polymerization of HEMA [13–16]. Since it is sensitive to light [34] and may undergo photodegradation during irradiation with the visible light, it was felt necessary to examine if any photoproducts of RF are formed during the irradiation of HEMA (up to 60 s). The TLC of the aqueous solutions of RF (1 × 10^−5^ M) used in the photolysis of HEMA, exposed to light for 5 min (solvent systems a and b, Section 2), indicated the presence of lumichrome (LC) at pH 9.0 only. In the alkaline solutions, the photodegradation of RF is greater than that in the acid region [20, 34]; however, under the present irradiation conditions (up to 60 s), no photoproducts of RF were detected in the irradiated solutions. The photoproducts of RF observed in aqueous solution are known and have previously been reported [20, 34–36].

### 3.2. Spectral Characteristics of Photolysed Solutions of RF

The spectral characteristics of the photolysed solutions of RF (pH 9.0) during irradiation (up to 60 s) showed no loss of absorbance at 444 nm in the visible region indicating the photostability of RF during the period. The formation of LC and other photoproducts of RF [20, 32, 37] would alter the spectral characteristics due to loss in concentration. Since RF has been used at a low concentration (1 × 10^−5^ M) in the polymerization of HEMA [13], its stability during the reaction is necessary to maintain its efficacy as a photoinitiator.

### 3.3. Assay of HEMA

 A UV spectophotometric method has been used for the assay of HEMA at 208 nm (pH 7.0) during the polymerization reactions. This wavelength corresponds to the absorption maximum of HEMA involving the *π*-π* transition (Table 1) and on appropriate dilution would give the concentration of HEMA monomer during the reaction. The validity of Beer's Law was confirmed in the desired concentration range prior to the assay, and the content of HEMA in polymerized solutions was determined using 7980 M^−1^ cm^−1^ as the value of molar absorptivity at the analytical wavelength (Table 1). The reproducibility of the method was confirmed by the assay of known amounts of HEMA in the concentration range likely to be found in polymerized solutions. The values of RSD for the assay indicate the precision of the method to be within ±3%. This is a new, rapid, and convenient method for the determination of HEMA in polymerized solutions since most of the previous workers have used dilatometric method for this purpose [13–15]. In some cases, the rates of polymerization of HEMA have been measured using gas chromatography [38], Raman spectroscopy [28], ATR-FTIR spectroscopy [29], and differential scanning calorimetry [39, 40]. The determination of HEMA has only been carried out during the initial stages of the reaction (~5% conversion) assuming that in this period a negligible change in volume would occur in the medium which does not affect the accuracy of the assay method with its defined reproducibility. RF at the dilutions used for the assay of HEMA in polymerized solutions (more than 100 fold) exhibits negligible absorbance at the analytical wavelength and hence does not interfere with the assay of HEMA.

### 3.4. Kinetics of Polymerization of HEMA

An approximate measure of the kinetics polymerization at low conversion of HEMA has been studied spectrophotometrically as described above. The assay data on HEMA during the initial stages of the reactions (~5% conversion) were subjected to an approximate kinetic treatment, and the photolysis of HEMA (a measure of polymerization) was found to follow an apparent first-order kinetics in the presence of various TEOHA concentrations (0.0025–0.0100 M). The polymerization of HEMA at low conversion remains homogenous since the polymer remains soluble in monomer-rich aqueous solutions [23]. The steady-state assumption of the rate of initiation being equal to the rate of termination is considered valid only at a low conversion of monomer [39, 41], depending upon the intensity of the radiation source (very low in this study see Section 2), and is represented by the apparent first-order rate constant (*k*_obs_) in this study. The kinetic treatment for the steady state assumption of polymerization reaction has been presented by Watts [24]. During the initial stages of the reaction (within ~5% conversion), the average degree of polymerization as well as the viscoelastic properties of HEMA would almost be constant on changing the coinitiator concentration (0.0025–0.0100 M). Under these conditions, the shrinkage properties of the polymerized solution would not be affected.

### 3.5. Effect of pH

**Figure 1:**
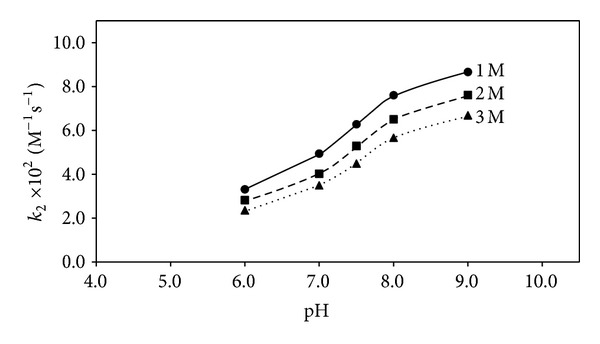
Rate-pH profiles for the polymerization of HEMA in presence of RF/TEOHA. HEMA concentration: (*⚫*) 1.0 M, (■) 2.0 M, (▲) 3.0 M.

The values of the determined *k*_obs_ for the polymerization of HEMA indicate that the rate of reaction is enhanced with an increase in pH from 6.0–9.0. Since the polymerization of HEMA has been carried out in the presence of TEOHA, a change in the ionization of TEOHA (p*K*_a_ 7.82) [42] from 98.5 to 6.1% at pH 6.0–9.0 would affect the rate of polymerization since it would facilitate electron transfer from TEOHA to RF triplet state [14] resulting in the greater polymerization of HEMA. The rate-pH profiles for the polymerization of HEMA at 1–3 M concentrations are shown in Figure 1. These indicate an enhancement in the rate, with pH, in the range of 6.0–9.0, as observed earlier by Valdebenito and Encinas [27]. This increase in the rate of polymerization, with pH, is attributed to the presence of a labile proton on the hydroxyl group of HEMA [39]. The relative decrease in the rate of polymerization of HEMA (6.0–9.0) from 1 to 3 M appears largely due to the viscosity effect as discussed below.

### 3.6. Effect of Viscosity

**Figure 2:**
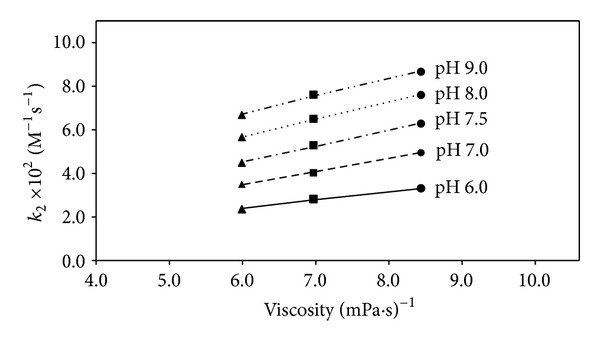
Plots of *k*_2_ for the polymerization of HEMA (1–3 M) in presence of RF/TEOHA versus inverse of solution viscosity. Symbols are as in Figure 1.

 It has been observed that the rate of polymerization of HEMA in aqueous solution (pH 6.0–9.0) is affected by a change in the viscosity of the medium on increasing the HEMA concentration (1–3 M). The plots of *k*_obs_ at different HEMA concentrations versus the inverse of viscosity of the solutions in the pH range 6.0–9.0 are linear (Figure 2), indicating that an increase in viscosity leads to a decrease in the rate of polymerization of HEMA probably as a result of the flavin excited singlet and excited triplet states quenching with an increase in viscosity [17]. The rate constants for diffusion controlled processes are a function of solvent viscosity [43]. Therefore, the rate of reaction would be controlled by solute diffusion and hence the degree of redox reactions occurring between the species involved in a particular medium. A small change in the viscosity of HEMA solutions with an increase in pH has been reported [27]. Thus, the HEMA concentration appears to be one of the controlling factors in the rate of polymerization.

### 3.7. Effect of TEOHA Concentration

TEOHA plays an important role in the polymerization of HEMA, and the reaction does not occur in the absence of the coinitiator. RF radicals interact with TEOHA to initiate the polymerization reaction. It has been reported that the rate of polymerization of HEMA is maximum in the presence of 0.01 M TEOHA [14]. In order to observe the effect of TEOHA concentration (0.0025–0.0100 M) and its interaction with HEMA in the initial stages of polymerization, the *k*_obs_ values were determined for the reactions carried out at various TEOHA concentrations. A plot of *k*_obs_ versus TEOHA concentrations was found to be linear and the slope yielded the second-order rate constant (*k*_2_) for the interaction of TEOHA with HEMA. The *k*_2_ values (Table 2) have been found to increase with an increase in pH as explained above for the reactions at pH 6.0–9.0. Thus, the rate of polymerization is dependent on the reactivity of the amine radicals produced during the reaction.

### 3.8. Mechanism of HEMA Polymerization

**Figure 3:**
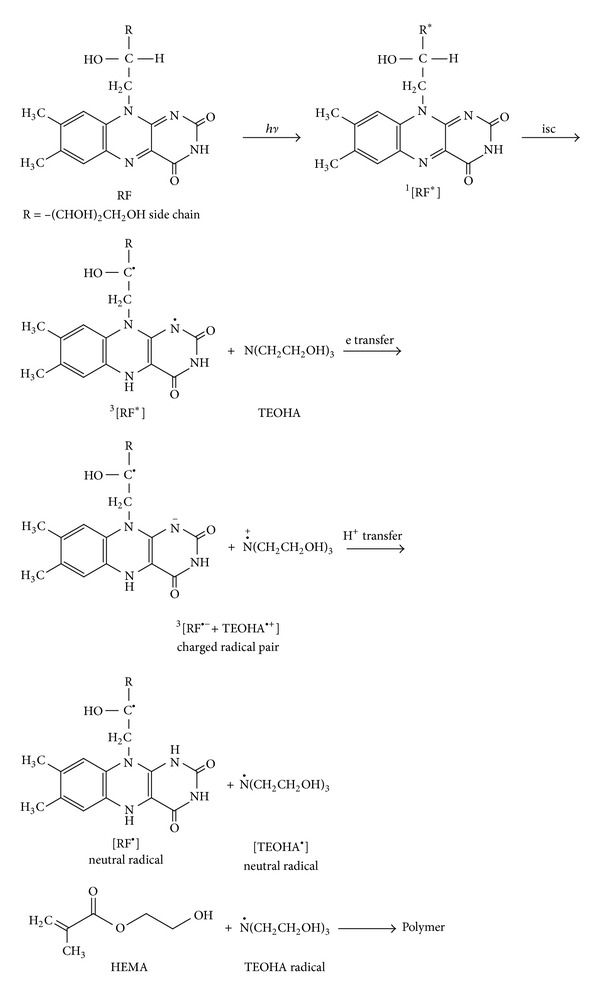
Scheme for the polymerization of HEMA initiated by RF/TEOHA in aqueous solution.

 A photoinitiation mechanism of HEMA polymerization by RF/TEOHA in aqueous solution has been proposed by Orellana et al. [14]. It has been suggested that the free radicals produced in the photoinduced electron transfer from TEOHA to excited RF lead to the polymerization of HEMA. A similar mechanism for HEMA polymerization photoinitiated by RF in the presence of amines in aqueous solution has been suggested by Encinas and Previtali [12]. The free radicals may also be produced from other photoinitiators such as ketones and add to HEMA to initiate the polymerization of the monomer [43]. Based on the mechanisms proposed by previous workers [12, 14], a more elaborate scheme involving the structural considerations of the species participating in the polymerization process is presented in Figure 3.

The photoinitiator, RF, on the absorption of visible light is promoted to the excited singlet state [^1^RF*] and by intersystem crossing (isc) to the excited triplet state [^3^RF*]. An electron transfer from TEOHA to [^3^RF*] leads to the formation of [RF^•−^] anion and [TEOHA^•+^] cation radical pair. This is followed by rapid proton transfer between the species leading to the formation of [RF^•^] and [TEOHA^•^] free radicals. The [TEOHA^•^] free radical interacts with HEMA and is added to the monomer double bonds. This would initiate the polymerization process and thus lead to the formation of the polymer. The rate and extent of HEMA polymerization would depend on factors such as RF and TEOHA concentrations, pH, and viscosity of the medium and light intensity and wavelengths of irradiation.

## 4. Conclusion

The polymerization of 1–3 M HEMA in aqueous solutions initiated by RF in the presence of TEOHA has been studied spectrophotometrically by its loss of absorbance at 208 nm. The rate of the reaction is increased with pH in the range of 6.0–9.0. In the initial stages of the reaction (~5% HEMA conversion), the monomer undergoes change by an approximate first-order kinetics, and the rate is dependent on TEOHA concentration. The second-order rate constants for HEMA-TEOHA interaction decrease with an increase in HEMA concentration as a result of higher viscosity and lower reactivity of the flavin radicals. The apparent first-order rate constants for HEMA polymerization are a linear function of the inverse of viscosity at pH 6.0–9.0, indicating a decrease in the rate of the reaction with an increase in the viscosity of the medium.

## Figures and Tables

**Table 1:** Calibration data for HEMA showing linear regression analysis^a^.

*λ*_max⁡_	208 nm
Concentration range	0.1–1.0 × 10^−4^ M
Slope	7980
SE (±) of slope	0.0112
Intercept	0.0010
Correlation coefficient	0.9995
Molar absorptivity (*ε*)	7980 M^−1^ cm^−1^

^a^Values represent a mean of five determinations.

**Table 2:** Second-order rate constants (*k*_2_) for the interaction of TEOHA with HEMA at pH 6.0–9.0^a,b^.

pH	*k*_2_ × 10^2^ M^−1^ s^−1 ^±S.D		
	Monomer : water ratio (1.21 : 8.79, v/v, 1.0 M)	Monomer : water ratio (2.42 : 7.58, v/v, 2.0 M)	Monomer : water ratio (3.63 : 6.37, v/v, 3.0 M)
6.0	3.32 ± 0.26	2.83 ± 0.22	2.36 ± 0.18
7.0	4.95 ± 0.35	4.03 ± 0.34	3.50 ± 0.28
7.5	6.29 ± 0.41	5.30 ± 0.40	4.49 ± 0.37
8.0	7.61 ± 0.62	6.51 ± 0.52	5.67 ± 0.45
9.0	8.67 ± 0.65	7.62 ± 0.54	6.69 ± 0.47

^a^ *N* = 3.

^b^Experimental conditions. Concentration of HEMA 1–3 M, concentration of TEOHA 0.0025–0.0100 M; wavelength visible radiation; exposure time 60 s; temperature 25 ± 1°C.
